# JUUL preference among Korean adult tobacco users and its effect on attempts to quit tobacco: A follow-up survey four months post JUUL launch

**DOI:** 10.18332/tid/160163

**Published:** 2023-03-14

**Authors:** Ju Sam Hwang, Kiheon Lee, Choon-Young Kim, Heejin Kim, Sungroul Kim, Cheol M. Lee

**Affiliations:** 1 Department of Family Medicine, Seoul National University Hospital, Seoul, Republic of Korea; 2 Department of Family Medicine, Seoul National University Bundang Hospital, Seongnam, Republic of Korea; 3 Department of Family Medicine, Seoul National University College of Medicine, Seoul, Republic of Korea; 4 Department of Family Medicine, Healthcare System Gangnam Center, Seoul National University Hospital, Seoul, Republic of Korea; 5 Department of Epidemiology and Health Promotion, Institute for Health Promotion, Graduate School of Public Health, Yonsei University, Seoul, Republic of Korea; 6 Department of Environmental Health Sciences, Soonchunhyang University, Asan, Republic of Korea

**Keywords:** tobacco, electronic nicotine delivery systems, JUUL, population surveillance, government regulation

## Abstract

**INTRODUCTION:**

This study examined the various factors in the selection of JUUL (and/or), a pod-mod type electronic cigarette (EC), and the changes in EC patterns before and after the JUUL debut on 24 May 2019, using follow-up survey data of adult tobacco users in South Korea.

**METHODS:**

This study examined transition outcomes among tobacco users and factors associated with future JUUL use. Convenience sample data were collected from adult tobacco users in South Korea, from March–April 2019 (baseline, n=2173) to September 2019 (follow-up, n=779). Results were obtained from the 779 respondents in the follow-up survey, and user data of one or more tobacco products were analyzed. The changes in the proportion of EC and JUUL use during this period were calculated, and multivariate logistic regression analysis was conducted to investigate the selection factors of JUUL.

**RESULTS:**

Four months after the JUUL launch, the proportion of current EC and JUUL users among the whole sample increased by 10.3% (42.6–52.9%, p<0.001) and 17.7 % (4.0–21.7%, p<0.001), respectively, while the proportion of triple users doubled (18.7% vs 37.5%, p<0.001). Among current EC users, the percentage of quitting EC within one month decreased from 18.7% to 8.7%; this change was more pronounced among concurrent JUUL users than non-JUUL users (p<0.001). In the multivariate logistic analysis with adjustment for possible confounders, JUUL use was significantly associated with male sex, young and middle age, and metropolitan residency status at the baseline survey.

**CONCLUSIONS:**

After the launch of JUUL in South Korea, EC users, including JUUL and triple users, increased significantly, but the intention to stop EC decreased significantly. Given the serious interests of the tobacco industry in these products, additional regulation is warranted.

## INTRODUCTION

Electronic cigarettes (ECs) are battery-powered devices that heat a solution, usually containing nicotine and various flavors, to be inhaled by users. The use of EC has risen dramatically in recent years, especially among adolescents and young adults^1^, leading to conflicts over how ECs are regulated in the public health community^2^. EC use can cause nicotine addiction and could give rise to starting conventional cigarette use. In contrast, EC use is likely far less harmful than conventional combustible cigarette (CC) use^3,4^. While ECs have been marketed as healthier alternatives to conventional CC smoking or smoking cessation aids, they have been primarily used in conjunction with CCs (dual use) instead of being used exclusively.

Largely, major concerns about ECs were elicited because of the rapid increase in the use of JUUL^5-7^; a 4th generation EC was first introduced to the US market in 2015. Four years after its launch, JUUL accounted for more than 75% of the total EC market^8^. JUUL uses a nicotine-based e-liquid to deliver a much higher nicotine concentration in a shorter time than the previous generation of ECs^8^. The JUUL-pod nicotine concentration of 5% (59 mg/mL) is marketed as being equivalent to 1 pack of cigarettes. Also, it is notable for its compact, sleek, and sophisticated design, as well as its appealing flavors^1,9,10^.

JUUL was launched in South Korea on 24 May 2019, and was of great interest to smokers. Before its launch, the EC usage rate was less than 4% for both adults and adolescents, which was much lower than the prevalence of current CC users^11^.

Tobacco companies consider South Korea an ideal test bed for new tobacco products. The number of adult male smokers in South Korea is the eighth largest of all OECD (Organization for Economic Cooperation and Development) member countries in 2019^12^. South Korea is known for its high population density which is more than ten times the global average^13^. Seoul, the capital of South Korea, where this study was conducted, ranked fourth in the world in gross domestic product (GDP) in 2018^14^. The large economy of South Korea makes it lucrative as a tobacco test-bed, as it is possible to analyze a population response to new products economically and efficiently. Tobacco companies launched heated tobacco products (HTPs) in Japan and South Korea as their test markets in 2017^15,16^. Based on these circumstances, South Korea could have had an advantage in efficiently examining consumer responses to new products, and hence JUUL was launched with extensive marketing in South Korea on 24 May 2019, following its success in the US market.

Multiple studies on JUUL use exist; previous studies have reported factors associated with JUUL use and predictors of smoking reduction and cessation^1,17-21^. A national study that was representative of the US revealed the relationship between JUUL use and nicotine dependence^22^. Additionally, other studies have reported that JUUL users were more common among the following demographics: young age, male sex, and higher socioeconomic status^17,21^. In the Assessment of the post-College Experience (ACE) cohort study data, ever and current users of JUUL significantly increased in just six months. Also, current JUUL use is related to JUUL advertisement exposure and perception of the harmfulness of JUUL^18^. While there has been a literature review on JUUL use in other countries, research on JUUL use in South Korea is limited.

In South Korea, it was possible to analyze the consequences of launching JUUL, as it debuted as an established EC product. The objectives of this study were to analyze the surveys administered before and after the debut of JUUL and include the following:

1. Describe the impact of JUUL’s launch among current tobacco users depending on each type of tobacco product;
2. Identify the changes in the EC use pattern and compare them among current EC users (between JUUL users and other EC users); and
3. Examine the baseline factors associated with subsequent JUUL use.

## METHODS

### Study participants

To investigate the usage patterns of new tobacco products, such as ECs and HTPs in South Korea, we used data from follow-up research from the THINK (Tobacco and Health IN Korea) study funded by the Korea Centers for Disease Control and Prevention^23^. In a baseline survey, convenience sampling of adults aged 19–64 years was conducted to account for the low prevalence of exclusive EC product usage in some general population groups.

Individuals who currently use more than one type of tobacco product among CCs, ECs or HTPs were included in this study. To investigate the changes in general adult tobacco use behavior and nicotine dependence, participants were recruited except for: those aged <19 years, pregnant women, nicotine replacement therapy (such as nicotine patches, gum, and lozenges) users, and severely ill patients with lung disease.

The THINK study participants recruited voluntary applicants from hospitals, universities, and Gallup Korea (online and offline: http://www.gallup.co.kr/)^23^. Among the 3004 participants, 2173 were from online and offline surveys conducted by Gallup Korea from 19 March to 17 April 2019. Of the 2173 participants, 779 were follow-up survey respondents (response rate: 35.8%) who completed the baseline online survey and consented to the follow-up survey [partial respondents (n=393), persons who refuse to respond (n=5), non-contact (n=992), and answered erroneously (n=4)] (Supplementary file Figure 2). The online follow-up survey was conducted from 2 to 17 September 2019, approximately four months after the launch of JUUL in South Korea. All participants received a financial incentive of 3000 South Korean Won (about 2.5 US$), each time they completed the baseline and follow-up THINK study surveys.

### Measures

*Types of tobacco use and behaviors*

Questions were asked to determine the past, ever, and current use of CCs, ECs, and HTPs. Current smokers were defined as those who answered that they smoke daily or intermittently (with more than 100 cigarettes used in their lifetime). Current EC users were defined as those who stated that they had used an EC in the past 30 days. Current HTP users were defined as individuals who reported using HTPs every day or within the previous 30 days of filling in the questionnaire.

The participants were allowed to respond to questions related to multiple products (e.g. CC, EC, or HTP). Current single-product usage was defined as the use of only one type of tobacco product (e.g. exclusive CC users) and defined as exclusive use. Current dual-product usage was defined as the use of two types of tobacco products (e.g. CC + EC, EC + HTP, or CC + HTP) and defined as dual use. Triple-product usage was defined as the use of three types of tobacco products (e.g. CC + EC + HTP). We categorized current tobacco users into seven groups according to their combinations of each product: exclusive users (CC, EC, or HTP), dual users (CC + EC, EC + HTP, or CC + HTP), and triple users (CC + EC + HTP).

Since HTPs are classified as ECs (cigarette-type ECs) by the South Korean government, confusion regarding the same may often arise among Koreans. Therefore, we have added detailed descriptions and photos of EC and HTP, along with the specific brands sold in South Korea to avoid misclassification (Supplementary file Figure 1).

Detailed EC use behavior was also assessed. To identify specific EC devices, we asked the current EC users the following questions: ‘Which EC device do you mainly use? Please select all device brands you are currently using’. There were 24 EC devices which were divided into closed system vaporizer (CSV) or rechargeable types. Multiple answers to each question were possible (detailed EC brand names and information are given in Supplementary file Table 1). In the baseline and follow-up surveys: ECs included JUULs and other EC device brands; vaping included JUUL use and other EC device use; EC users who currently use JUUL were classified as JUUL users; other EC users were categorized as non-JUUL users.

For assessment of dependence, the time to first vaping (TTFV) item was also used to measure nicotine dependence, as in previous studies^24,25^. The answers to the question ‘How soon after waking up do you vape in the morning?’ were divided into two groups: within 30 min (higher nicotine dependence) and more than 30 min (lower nicotine dependence). Willingness to quit vaping (EC use) was evaluated using the following question of the transtheoretical model (TTM): ‘Do you have any plans to quit ECs next month?’. Participants planning to quit EC use within one month of the survey were categorized as being at the ‘preparation stage’ and the rest as ‘others’^26^. Regarding tobacco cessation attempts, participants who used these products were asked individually whether they had tried quitting each product (up to three types of tobacco products) last year.

*Covariates*

The following sociodemographic and economic characteristics were measured: age group (19–39, 40–49, and ≥50 years), sex (female or male), monthly household income [≥5 million South Korean Won (comparable to ≥US$4000), or <5 million Won], occupation (clerical workers, sales and service workers, or unemployed), residential area (metropolitan, other city or province), education level (less than high school, Bachelor’s degree, graduate degree or higher), and marital status (single, married, divorced, separated, or widowed). Additionally, several lifestyle and health-related factors were assessed.

Hypertension was defined as the use of medication for hypertension, diabetes as the presence of hypoglycemic agents, dyslipidemia as the use of lipid-lowering medications, and coronary heart disease (CHD) as having been diagnosed with myocardial infarction or unstable angina. CHD, stroke, and cancer were defined as previously diagnosed, while chronic cough was defined as a cough lasting more than three months within a year of the baseline survey.

### Statistical analysis

We conducted a Z-test to assess whether the current EC user groups differed for some categorical variables, such as EC use frequency, TTFV, and willingness to quit vaping, and how these proportions changed from baseline to the follow-up survey. Multivariate logistic regression models were employed to investigate the baseline characteristics, such as age, sex, monthly household income, occupation, and residency status, education level, chronic cough, tobacco type of use, and plan to quit tobacco use within the next month associated with the future JUUL use, while controlling for sociodemographic and economic factors and tobacco use behaviors. The STATA statistical software (version 16.0; StataCorp., College Station, Texas, USA) was used for statistical analyses, and p<0.05 was considered statistically significant.

## RESULTS

Table 1 summarizes the baseline characteristics of the 779 respondents included in the final analysis. The majority of respondents were male (79.6%), clerical workers (71.9%), and metropolitan residents (70.1%), and most had obtained a college degree (87.4%). The most common tobacco products being used were CCs (71.8%), HTPs (56.7%), and ECs (42.6%) (multiple responses were possible). Additionally, of the respondents, 54.4% reported attempting to quit CCs, and 47.3% answered attempting to quit ECs in the past year.

**Table 1:** Baseline general characteristics of study participants, South Korea 2019 (N=779)

*Characteristics*	*All participants n (%)*
**Age** (years), mean	43.9
19-39	262 (33.6)
40–49	292 (37.5)
≥50	225 (28.9)
**Sex**	
Male	620 (79.6)
Female	159 (20.4)
**Residence**	
Metropolitan	546 (70.1)
Other areas	233 (29.9)
**Education level**	
High school or lower	98 (12.6)
Bachelor’s degree	580 (74.4)
Graduate degree or higher	101 (13.0)
**Average monthly household income** (US$)^a^	
<4000	368 (47.2)
≥4000	411 (52.8)
**Occupation^b^**	
Clerical workers	560 (71.9)
Sales and service workers	169 (21.7)
Unemployed	50 (6.4)
**Marital status**	
Single	230 (29.5)
Married	527 (67.7)
Divorced /separated	19 (2.4)
Widowed	3 (0.4)
**Alcohol use frequency**	
1 per month	183 (23.6)
2–4 per month	298 (38.2)
Weekly at least	298 (38.2)
**Health status**	
Hypertension	118 (15.2)
Diabetes	53 (6.8)
Dyslipidemia	81 (10.4)
Coronary heart disease	28 (3.6)
Stroke	23 (2.9)
Cancer	27 (3.5)
Chronic cough	72 (9.2)
**Subjective health perception**	
Good	222 (28.5)
Fair	453 (58.1)
Poor	104 (13.3)
**CC use status**	
Never	17 (2.1)
Former	203 (26.1)
Current	559 (71.8)
**EC use status**	
Never	315 (40.5)
Former	132 (16.9)
Current	332 (42.6)
**HTP use status**	
Never	255 (32.8)
Former	82 (10.5)
Current	442 (56.7)
**Past year tobacco quit attempts**	
Quit CCs	304 (54.4)
Quit ECs	157 (47.3)
Quit HTPs	176 (39.8)
**EC harm perception^c^**	
Less harmful than CCs	183 (23.5)
Similar as CCs	273 (35.0)
Not less harmful than CCs	323 (41.5)
**EC use for smoking cessation perception^d^**	
Positive	174 (22.3)
Neutral	249 (32.0)
Negative	356 (45.7)

CC: combustible cigarette. EC: electronic cigarette. HTP: heated tobacco product.

a Converted 5 million South Korean Won to US$4000.

b Clerical workers include management, clerical and financial work, research and technical engineering jobs, education, law, police service, health and medical work, art, and design work. Sales and service workers include military service, accommodation, food and cleaning industries, production and sales, and construction workers. Unemployed includes not working for more than an hour for income purposes in the previous week.

c EC harm perception: Responses to the question, ‘Do you think electronic cigarettes are less harmful to your health than combustible cigarettes?’.

d EC for smoking cessation: Responses to the question, ‘Do you think electronic cigarettes are helpful for smoking cessation?’.

### Changes in tobacco use behaviors after the launch of JUUL

**Figure 1:**
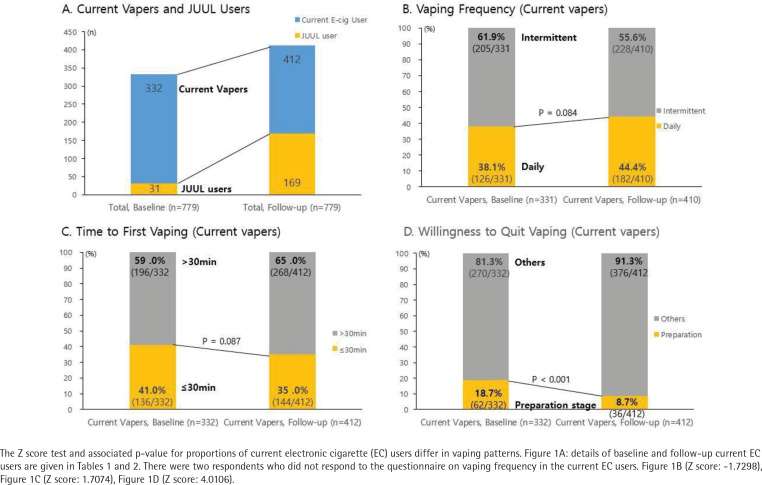
Transition of the number of JUUL users and vaping patterns (vaping frequency, time to first vaping, intention to quit vaping) from pre- to post JUUL launching

Table 2 shows the transition of the participants’ tobacco usage from before to after JUUL’s release. At baseline, among all the tobacco users, 47.6% were exclusive users, 33.7% were dual users, and 18.7% were triple product users. After JUUL’s launch, the number of any current CC users, including exclusive, dual, and triple users increased by 17.5% (Table 2), the number of any current EC users increased by 10.3% (Table 2, Figure 1A), and the number of any current HTP users increased by 3.2% (Table 2), while the number of triple users doubled. In contrast, the number of exclusive EC or exclusive HTP users decreased sharply.

**Table 2:** The transition of participants’ tobacco use status from pre- to post JUUL launching in South Korea (N=779)

	*Baseline n (%)*	*Follow-up n (%)*	*Change n (%)*
**Exclusive use**			
CC	207 (26.6)	202 (25.9)	-5 (-2.4)
EC	43 (5.5)	16 (2.1)	-27 (-62.8)
HTP	121 (15.5)	42 (5.4)	-79 (-65.3)
**Dual use**			
CC + EC	87 (11.2)	86 (11.0)	-1 (-1.1)
EC + HTP	56 (7.2)	18 (2.3)	-38 (-67.9)
CC + HTP	119 (15.3)	115 (14.8)	-4 (-3.4)
**Triple use**			
CC + EC + HTP	146 (18.7)	292 (37.5)	146 (100)
**Tobacco use status**			
Quit tobacco^a^	0	8 (1.0)	
Any CC use	559	695	136 (17.5)
Any EC use	332	412	80 (10.3)
Any HTP use	442	467	25 (3.2)

CC: combustible cigarette. EC: electronic cigarette. HTP: heated tobacco product.

a Did not use any tobacco (CC, EC or HTP) in the follow-up survey.

We compared the changes in EC use behaviors before and after the launch of JUUL in South Korea (Figure 1). The proportion of current JUUL uses increased more than 5-fold from the baseline to the follow-up survey (4.0–21.7%, p<0.001). Among the current EC users, the proportion of JUUL users more than quadrupled (9.3–41.0%), and the proportion of those willing to quit vaping was more than half (18.7–8.7%, p<0.001). In addition, the proportion of daily EC users increased (38.1–44.4%, p=0.084), and the number of current EC users with higher nicotine dependence (TTFV ≤30 min) decreased (41.0–35.0%, p=0.087).

**Figure 2:**
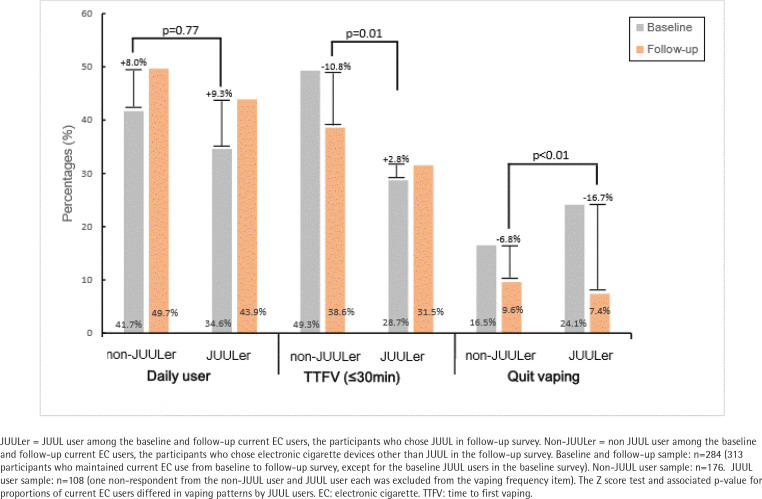
Comparison of vaping patterns between baseline and follow-up survey by follow-up JUUL user

We also compared the changes in EC use behaviors between JUUL users and non-JUUL users (Figure 2). Of the participants who maintained current EC use from the baseline to the follow-up survey (n=313), those using a JUUL device (n=29) in the baseline survey were excluded, and 284 participants were enrolled in the sensitivity analysis. Specifically, those who reported using JUUL at baseline (n=29) were excluded from the analysis of factors related to the initiation of JUUL use. JUUL was officially released in South Korea on 24 May 2019; therefore, it can be inferred that 29 participants purchased JUUL through an illegal market before its official release in South Korea. Assuming that 108 participants purchased JUUL in the official tobacco market in South Korea, these participants started using JUULs after the baseline survey but before the follow-up survey began. In this sensitivity analysis group (n=284), 38.0% were JUUL users (n=108), and the rest were non-JUUL users (n=176). As for willingness to quit vaping, compared to non-JUUL users, the decrease in the ratio of JUUL users was greater in the follow-up survey (-16.7% vs -6.8%, p<0.01). In addition, we perceived a difference in the nicotine dependence between JUUL users and non-JUUL users with nicotine cravings (i.e. EC use within 30 min of waking) (2.8% vs -10.8%, p=0.01). The proportion of daily EC use was not significantly different between the groups (9.3% vs 8.0%, p=0.77).

### Factors associated with selecting JUUL in the follow-up survey

Of the current tobacco product users who did not use JUUL at baseline (n=748), 153 (20.4%) were identified as new JUUL users in the follow-up survey. The mean age of new JUUL users was 41.3 years; 81.0% (n=124) of them were men, 87.6% (n=134) were metropolitan residents, and 83.7% (n=128) did not report chronic cough (Table 3). Among the 153 participants in the baseline survey, 72.6% were current CC users, 70.6% were current EC users, and 74.5% were HTP users.

**Table 3:** Multivariate logistic regression analysis examining baseline sociodemographic and tobacco use behavior by JUUL users in the follow-up survey (N=748)

	*Follow-up JUUL users (N=153) n (%)*	*AOR (95% CI)*	*p*
**Age** (years)			
Mean	41.3		
≤39	64 (41.8)	2.86 (1.26–6.48)	0.012
40–49	66 (43.1)	2.70 (1.19–6.14)	0.018
≥50 (Ref.)	23 (15.0)	1	
**Sex**			
Male	124 (81.0)	2.62 (1.37–4.98)	0.003
Female (Ref.)	29 (18.9)	1	
**Monthly household income** (US$)			
<4000 (Ref.)	56 (36.6)	1	
≥4000	97 (63.4)	1.62 (0.89–2.96)	0.113
**Occupation**			
Clerical workers	117 (76.5)	1.26 (0.57–2.78)	0.565
Sales and service workers (Ref.)	28 (18.3)	1	
Unemployed	8 (5.2)	1.29 (0.37–4.43)	0.691
**Residence**			
Metropolitan	134 (87.6)	4.20 (2.01–8.75)	<0.001
Other areas (Ref.)	19 (12.4)	1	
**Education level**			
High school or lower	95 (12.7)	1.30 (0.44–3.86)	0.637
Bachelor’s degree (Ref.)	558 (74.6)	1	
Graduate degree or higher	95 (12.7)	0.92 (0.41–2.07)	0.832
**Chronic cough**			
No (Ref.)	128 (83.7)	1	
Yes	25 (16.3)	1.21 (0.57–2.55)	0.620
**CC use status** (baseline)			
Never (Ref.)	6 (3.9)	1	
Former	36 (23.5)	0.38 (0.10–1.43)	0.153
Current	111 (72.6)	0.70 (0.20–2.42)	0.574
**EC use status** (baseline)			
Never (Ref.)	27 (17.6)	1	
Former	18 (11.8)	1.84 (0.62–5.51)	0.275
Current	108 (70.6)	0.97 (0.46–2.06)	0.943
**HTP use status** (baseline)			
Never (Ref.)	29 (19.0)	1	
Former	10 (6.5)	1.01 (0.28–3.66)	0.984
Current	114 (74.5)	1.19 (0.48–2.95)	0.715
**Plan to quit CC within next one month** (baseline)			
No (Ref.)	132 (86.3)	1	
Yes	11 (7.2)	1.81 (0.41–8.10)	0.435
No response	10 (6.5)		
**Plan to quit EC within one month** (baseline)			
No (Ref.)	144 (94.1)	1	
Yes	9 (5.9)	0.99 (0.21–4.81)	0.995
No response	0		
**Plan to quit HTP within one month** (baseline)			
No (Ref.)	113 (73.9)	1	
Yes	8 (5.2)	0.41 (0.08–2.10)	0.285
No response	32 (20.9)		

AOR: adjusted odds ratio. CC: combustible cigarette. EC: electronic cigarette. HTP: heated tobacco product.

In our logistic regression analysis, we observed that future JUUL use was significantly associated with several baseline characteristics compared with non-JUUL use in the follow-up survey. Younger respondents, compared to those aged ≥50 years, were significantly associated with subsequent JUUL use: ≤39 years (AOR=2.86; 95% CI: 1.26–6.48); and 40–49 years (AOR=2.70; 95% CI: 1.19–6.14). Males (AOR=2.62; 95% CI: 1.37–4.98) and metropolitan residents (AOR=4.20; 95% CI: 2.01–8.75) were also associated with future JUUL use. Participants who had CC cessation plans within the next month (baseline survey) were not associated with future JUUL use (Table 3).

## DISCUSSION

Our results show that since JUUL was launched, the proportion of EC users increased by 10.3% among the tobacco product users [from 42.6% (332/779) to 52.9% (412/779)], and the proportion of current JUUL users among the current EC users more than quadrupled [from 9.3% (31/332) to 41% (169/412)] in number. The number of exclusive EC users decreased by 62.8% (43 to 16) and exclusive HTP users decreased by 65.3% (121 to 42), while the number of triple users increased by 100% (146 to 292).

In current vapers, there were no significant differences in nicotine dependence measured using vaping frequency and TTFV before and after the JUUL debut. However, the proportion of those willingness to quit vaping within one month significantly decreased by 10% (from 18.7 to 8.7%). Meanwhile, JUUL users showed increased nicotine dependence (based on TTFV) and decreased willingness to quit vaping compared to non-JUUL users. Figures 1 and 2 show that the willingness to quit vaping seemed to have decreased, with this change being more apparent in JUUL users than non-JUUL users (-16.7% vs -6.8%). Our findings support previous research findings that demonstrated a relationship between JUUL use and nicotine addiction in adults^27^. The association between JUUL use and nicotine dependence could vary, depending on circumstances, such as the willingness to quit smoking, e-liquid nicotine concentration, regulatory policy environments on EC sale, and tobacco users’ age group. Thus, research tailored to examine the complexities of novel EC brand preferences, nicotine content, and nicotine dependence is required.

This study also noted that male sex, younger age, and metropolitan residence were associated with future JUUL use. Our findings were consistent with other studies reporting JUUL usage was associated with male sex, younger age, and higher socioeconomic status. Case et al.^17^ reported that JUUL users versus other electronic nicotine delivery systems (ENDS) users were more likely to be male, younger, and of a higher socioeconomic status (SES), based on US college student data^17^. Vallone et al.^21^ suggested that factors such as younger age (18–20 years), combustible tobacco use, and low harm perception contribute to future JUUL use among e-cigarette-naïve participants in US representative longitudinal samples (aged 15–34 years).

Previous studies have addressed changes in use patterns among dual users of CCs and ECs and reported a relationship between the type of tobacco use and cessation. In the Population Assessment of Tobacco and Health (PATH) study, Waves 1 and 2 (2013–2015), EC use patterns were found to be highly variable over a 1-year period^28^. Approximately half of the adult EC users discontinued their EC use after one year. Among dual users in Wave 1, 44.3% continued dual use, and 43.5% and 12.1% remained exclusive CC and EC users, respectively. In our study, exclusive EC users or EC + HTP dual users decreased by -62.8% and -67.9%, respectively, in the follow-up survey; however, the number of CC + HTP dual users remained fairly steady (-3.4%). Furthermore, as the number of CC + EC + HTP triple users doubled in the follow-up survey, it could be inferred that those who used CC, such as in combinations of CC + EC, CC + HTP, and CC + EC + HTP, maintained their tobacco use patterns.

The willingness to quit vaping decreased among current EC users after the JUUL debut. Furthermore, follow-up JUUL users were less willing to quit vaping than non-JUUL users. Since the launch of JUUL in the US, it has been very popular among adolescents and young adults for many years. In this context, it is presumed that the willingness to quit vaping decreased because JUUL users’ tobacco product satisfaction is higher than that of other EC device users^27,29^.

Understanding the impact of JUUL’s debut on the tobacco market in South Korea can help us understand the demands of novel tobacco users. JUUL was popular in the Korean tobacco market during the early phase of its debut. The sales proportion of the CSV-type EC increased, accounting for up to 1.1% (9.8 million pods) of the total tobacco market share in the 3rd quarter of 2019^11^. However, as e-cigarette or Vaping Use-Associated with Lung Injury (EVALI) was first reported in August 2019^30^, the Ministry of Health and Welfare of South Korea has officially recommended the discontinuation of EC three times since September 2019. Subsequently, the sales proportion of the CSV type EC fell sharply, from 1.1% to 0.1% (0.9 million pods) of the total tobacco market sales in the first quarter of 2020. Moreover, after the introduction of JUUL, the HTP sales decreased from 11.5% (in the 2nd quarter) to 9.4% (in the 3rd quarter). However, it increased to 10.3% of the total tobacco market sales in the first quarter of 2020^11^. These two tobacco products appear to have been consumed as alternatives in South Korea.

The observed poly-use patterns associated with novel tobacco products, such as ECs and HTPs, suggest that the dual or triple use may continue rather than only in the transition period before quitting smoking^31-33^. Until now, the tobacco industry has continued to develop next-generation tobacco products to expand its market^34,35^. This has sparked concerns regarding public health and tobacco control in terms of nicotine addiction and its potential health risks.

### Strengths and limitations

This study has several limitations that need to be acknowledged. We analyzed a convenience sample from the THINK study. Therefore, the findings cannot be generalized to other countries because of the differences in tobacco use behaviors and restriction policies. However, it provided insight into how ECs affect the transition of tobacco market sales by type when JUUL is introduced to one nation. Especially in South Korea, HTPs accounted for most of the market sales of novel tobacco products, providing information on the transition in use patterns of novel tobacco products after the introduction of JUUL. Also, this study conducted a short-term follow-up survey after about four months; however, this longitudinal study highlighted the changes in the tobacco market immediately after the introduction of JUUL among current tobacco users in South Korea.

The factors associated with JUUL use, such as male sex, young age, and metropolitan residence, are concordant with the findings of other populations^17,21^. The ACE cohort study showed that the perception of JUUL and JUUL-related advertising exposure are meaningful factors for elucidating the smoking patterns in adults^18^. Regrettably, we did not include questions on the frequency of EC use per day and e-liquid concentration, thus limiting the analysis of nicotine dependence.

In 2019, the smoking rate of South Koreans, reported on an international basis, was 16.4%, while the cigarette usage rate of Korean adult men was 35.7%, which was higher than that of women (6.7%). The current (daily or intermittent) rate of EC use in adults is 17.8% for men and 3.9% for women, and there is a gender difference in tobacco usage rate in South Korea. This study used a convenience sample wherein 79.6% of the study population were men. Therefore, considering the circumstances in which our study’s results were male-focused, this sex-skewed results are understandable.

Our study has several strengths. For a more accurate analysis of the impact of JUUL introduction on tobacco users, the study excluded baseline JUUL users (n=31) and examined the factors associated with subsequent JUUL use and changes in EC use patterns before and after the JUUL launch. As 779 participants used at least one type of tobacco (CCs, ECs, or HTPs) in the baseline survey, they could provide information on the use of exclusive, dual, and triple use, including JUUL. Furthermore, pictures of HTP and EC devices, including JUUL, were presented in the questionnaires to enhance the participants’ understanding and the questionnaire’s accuracy. Further studies are required to examine the long-term effects of nicotine dependence and changes in tobacco use behavior and related health outcomes among novel tobacco product users.

## CONCLUSIONS

This study provided information about the changes in EC use patterns and predictors of JUUL use after a JUUL debut. Our findings suggest that JUUL use leads to a delay in EC users’ willingness to quit EC among Korean adult tobacco users.

## Data Availability

The data supporting this research cannot be made available for privacy or other reasons. The Korea Center for Disease Control and Prevention (KCDC) did not allow us to disclose a copy of this survey.
