# Supplementary material for: JUUL preference among Korean adult tobacco users and its effect on attempts to quit tobacco: A follow-up survey four months post JUUL launch
Source: Tob Induc Dis. 2023 Mar 14;21:39. doi: 10.18332/tid/160163 (PMC10012008; doi:10.18332/tid/160163)
Supplement: Supplementary file 1 [file TID-21-39-s1.pdf]

Supplementary Materials

Appendix Table 1. A Detailed List of Vaping Device brand with Website.

EC Device Brand	Baseline, n	Follow up. n	Category	Website
JUUL	31	169	CSV	https://www.juul.com/
Dubacco	55	42	Rechargable	https://dubacco.en.ec21.com/
Ramiya	12	9	Rechargable	http://www.ramiya.co.kr/ab-1854
Ebaco[a]	61	32	Rechargable	http://www.ebacomall.com 1)
Vogue	45	47	Rechargable	http://www.voguecigar.com
Haka	50	39	Rechargable	https://e-cig.co.kr/new/
Coco	21	19	Rechargable	http://www.etab.co.kr
Justfog	36	33	Rechargable	http://www.justfog.com
Ares	18	15	Rechargable	http://www.aidakorea.co.kr/
Gear mini	7	14	Rechargable	https://www.aidakorea.co.kr
Geekvape	5	8	Rechargable	https://www.geekvape.com/
Vaporesso	13	12	Rechargable	https://www.vaporesso.com/
Justone	17	21	Rechargable	http://www.justfog.com
V-park	9	9	Rechargable	https://www.ave40.com/
Vega	14	21	Rechargable	http://www.danielkorea.com
Viper	26	24	Rechargable	http://www.vipercig.com/
Cupti	4	4	Rechargable	https://www.directvapor.com/
Other pod-mod e-cigarettes not in the list	6	19	CSV	
Viento	7	14	CSV	http://vientokr.com
Phix	8	12	CSV	https://phixvapor.com
Vladdin	6	5	CSV	https://www.vladdinvapor.com
MI-POD	2	8	Rechargable	https://mipod.com
LABIT	2	8	CSV	http://labitec.kr
Etc.	7	16		
Total	464	606		

Abbreviations: EC, electronic cigarette; CSV, Closed System Vaporizer.

[a]Ebaco: Website was closed.
The e-cigarette brand mentioned randomly above had been sold in the tobacco retail shop in the South Korea, at the time of the survey (baseline and follow-up). Multiple responses were possible.

Appendix Figure 1. An introduction of the questions and images used to assess the type of tobacco products they used and its clarification are presented in below.

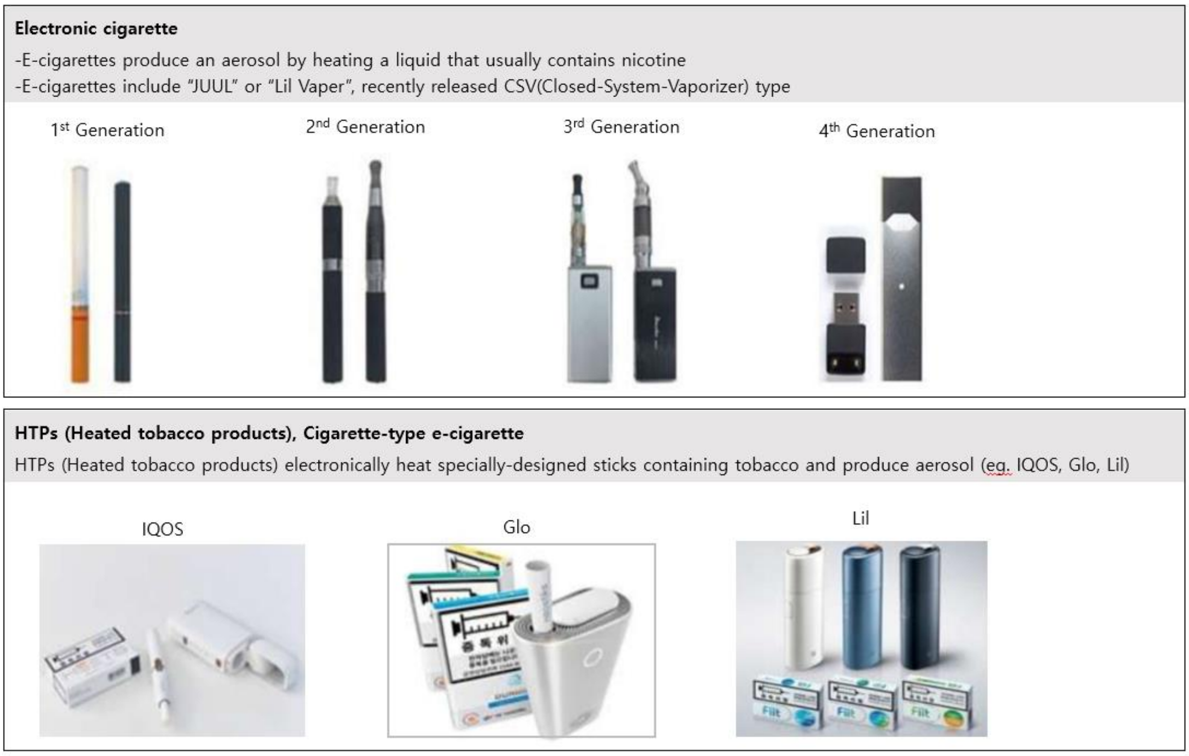

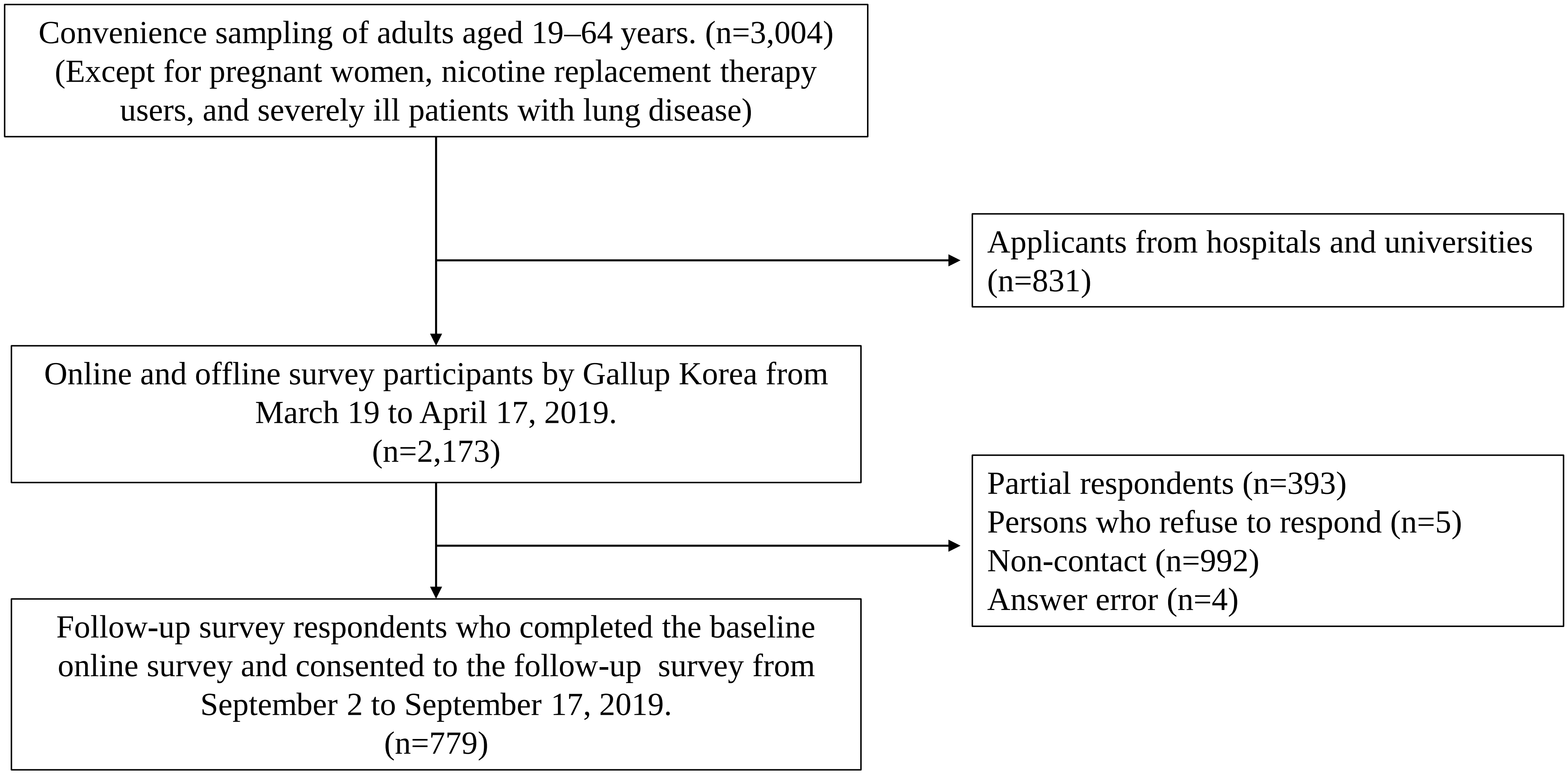

Appendix Figure 2. Flow chart of study participants.

© 2023 Hwang J.S. et al.
